# Could EBT Machines Increase Fruit and Vegetable Purchases at New York City Green Carts?

**DOI:** 10.5888/pcd14.170104

**Published:** 2017-09-21

**Authors:** Andrew Breck, Kamila Kiszko, Olivia Martinez, Courtney Abrams, Brian Elbel

**Affiliations:** 1 New York University Robert F. Wagner School of Public Service, New York, New York; 2 New York University School of Medicine, New York, New York

## Abstract

**Introduction:**

Residents of some low-income neighborhoods have limited access to fresh fruits and vegetables. In 2008, New York City issued new mobile fruit and vegetable cart licenses for neighborhoods with inadequate availability of fresh produce. Some of these carts were equipped with electronic benefit transfer (EBT) machines, allowing them to accept Supplemental Nutrition Assistance Program (SNAP) benefits. This article examines the association between type and quantities of fruits and vegetables purchased from mobile fruit and vegetable vendors and consumer characteristics, including payment method.

**Methods:**

Customers at 4 produce carts in the Bronx, New York, were surveyed during 3 periods in 2013 and 2014. Survey data, including purchased fruit and vegetable quantities, were analyzed using multivariable negative binomial regressions, with payment method (cash only vs EBT or EBT and cash) as the primary independent variable. Covariates included availability of EBT, vendor, and customer sociodemographic characteristics.

**Results:**

A total of 779 adults participated in this study. Shoppers who used SNAP benefits purchased an average of 5.4 more cup equivalents of fruits and vegetables than did shoppers who paid with cash. Approximately 80% of this difference was due to higher quantities of purchased fruits.

**Conclusion:**

Expanding access to EBT machines at mobile produce carts may increase purchases of fruits and vegetables from these vendors.

## Introduction

Cities in the United States are increasingly relying on mobile produce vendors to increase access to fresh fruits and vegetables (1–3). On March 13, 2008, New York City Mayor Michael Bloomberg authorized Local Law 9, establishing 1,000 permits for mobile fruit and vegetable vendors, called “Green Carts,” across areas that were deemed to have low food access (4). One goal of this program was to increase availability of fruits and vegetables in areas in which residents consume the least amounts of fresh produce (4,5) and have the highest obesity rates in the city (6).

As of 2013, there were nearly 500 active Green Cart vendors in the city, operating primarily in the Bronx and Manhattan (7). The New York City Department of Health and Mental Hygiene (NYC DOHMH) identified eligible Green Cart vending neighborhoods as those in which at least 14% of residents had reported not having consumed any fruits or vegetables the previous day (5). For many low-income families, financial constraints may prevent access to a nutritious diet, despite having fresh produce available for sale in the neighborhood. To address this barrier, the city facilitated a public–private partnership to provide financial support to eligible mobile produce vendors to cover the cost of electronic benefit transfer (EBT) machines that are necessary to accept Supplemental Nutrition Assistance Program (SNAP) benefits (8,9). As of 2013, 27% of vendors were equipped with EBT machines (7).

Research indicates that Green Carts typically locate in areas with existing commercial activity and high pedestrian traffic (6,10). Most customers are part of the target population: people with low income, members of minority groups, and residents of neighborhoods in which the carts are located. Furthermore, Green Cart customers shop at the carts regularly and report that since doing so they have increased their produce consumption (7). A NYC DOHMH evaluation of the Green Cart program from 2008 to 2011 also found that in precincts in which Green Carts were located, there was an increase in the proportion of other food retail outlets offering fresh produce, possibly because of an increase in consumer demand generated by the presence of the Green Carts (11). In our earlier work examining dollars spent per transaction at Green Carts in the Bronx, we found that customers spent on average a little more than $4 per transaction. Customers who paid using SNAP benefits spent more than $8 per transaction (12).

It remains unclear to what extent the Green Carts themselves assuage issues of low food access or facilitate healthy food purchases, especially among customers paying with SNAP benefits. In this study we built on our previous work and set out to answer 2 research questions: 1) What are the types and quantities of fruits and vegetables purchased at Green Carts in the Bronx, New York?, and 2) What consumer characteristics, including the use of SNAP, are correlated with these purchases?

## Methods

Data for this cross-sectional study were collected at 4 privately run and independently operated Green Carts in the Bronx, New York. This convenience sample of vendors was selected on the basis of referrals of Green Cart program–interested vendors from the NYC DOHMH. Each of these vendors generally operated between 7:30 AM to 7:00 PM, Monday through Friday, and sold a comparable variety of fresh produce items at similar prices. Customers, whom research assistants confirmed were aged at least 18 years, were invited to respond to a short survey (27 multiple-choice questions) about their food shopping and consumption patterns. Research assistants took an inventory of items each participant purchased during their Green Cart transaction. Interviews were completed in either English or Spanish, between 1:30 PM and 5:30 PM, Monday through Friday. Approximately 90 surveys were collected at each cart during each round of data collection. The overall survey response rate was 70%. Study participants received a single ride transit pass (value: $2.50) upon completion of the survey. This study was approved by the New York University School of Medicine Institutional Review Board. All participants provided verbal consent.

Research assistants interviewed customers at each cart during several periods: 1) June and July 2013, 2) September and October 2013, and 3) June and July 2014. Our original research plan included a 2 × 2 difference–in-difference design: surveying customers at 2 carts with and 2 without EBT in the pre-period, and 4 carts in the post-period, when all vendors were scheduled to have EBT machines. However, because of unanticipated changes in vendor operation and administrative delays in the distribution of EBT machines, data collection did not proceed as planned. Vendor A (observed during periods 1 and 2) acquired an EBT machine between visits. Vendors B (periods 1 and 3) and C (periods 1, 2, and 3) had EBT machines at each period of data collection. Finally, Vendor D (periods 1 and 3) did not have an EBT machine during either visit. Accordingly, we were unable to conduct our planned difference-in-difference analysis. Instead, we analyzed our data as a pooled cross-section and acknowledged the limitations of this design.

The types and quantity of fruit and vegetable purchases were entered into the Automated Self-Administered 24-hour Recall system (ASA24), a web-based program developed by the National Cancer Institute (13). On the basis of these entries, ASA24 calculated comparable food quantities (cup equivalents, which are a volume measure of the edible portion of the item that fits in a 1-cup measuring cup [14]) and detailed nutritional content estimates using the US Department of Agriculture’s Food and Nutrient Database for Dietary Studies (US Department of Agriculture, version 4.1). Research assistants completed data entry into ASA24 in February and March 2015.

We calculated summary statistics for both the full sample of respondents and the subsample of only the respondents surveyed at carts operating with an EBT machine. These descriptive statistics included frequencies (participant characteristics) and means (varieties and cup equivalents of fruits and vegetables purchased). Our primary outcome of interest — cup equivalents of fruits and vegetables — was nonnegative and positively skewed. Accordingly, we modeled this outcome using negative binomial regression, controlling for customer characteristics, including race/ethnicity (non-Hispanic white, non-Hispanic black, Hispanic, non-Hispanic other/refused), age in years (18–24, 25–39, 40–64, ≥65), sex (male, female), education level (high school degree or less, some college, bachelor’s degree or more, missing/refused), and annual household income (<$25,000; $25,000–$49,999; ≥$50,000; missing/refused) to calculate the relationship between payment method and the type (dark green, red or orange, starchy, or other vegetables) and quantity of fruits and vegetables purchased. Analyses were conducted using Stata v13 (StataCorp).

## Results

The full analytic sample included 779 customer surveys (Table 1). Most respondents were female (74%), Hispanic (54%), had no more than a high school degree (64%), and lived in households with an annual household income less than $25,000 (53%). Forty-two percent of respondents received SNAP benefits. Respondents were approximately equally as likely to report that they usually purchased fruits and vegetables at a grocery store (42%) as they were to report buying these items from a produce cart (45%). Most respondents paid for their purchase using only cash (87%) and reported that their purchase was to be shared (71%). Nearly 20% of respondents at EBT-equipped carts reported using SNAP benefits to pay for their purchase. Customers paying with SNAP were predominantly female (90%), Hispanic (75%), lived in households with less than $25,000 in annual household income (77%), and purchased items to share (84%). Most customers using SNAP benefits were unemployed (63%) versus slightly more than one-third (36%) of the full sample. Customers paying with SNAP benefits reported that their most common source for fruits and vegetables was produce carts (64%).

**Table 1:** Demographic and Purchase Descriptive Characteristics for Full and Subsamples of Survey Respondents at 4 Green Carts in New York City, 2013 and 2014

Characteristic	Entire Sample (n = 779), n (%)	Consumers at Green Carts With EBT (n = 516)		Consumers Who Made Purchases Using SNAP (n = 98)	
		n (%)	*P* Value^a^	n (%)	*P* Value^a^
**Payment method**					
Cash only	681 (87.4)	418 (81.0)	<.001	—	<.001
SNAP or SNAP and cash	98 (12.6)	98 (19.0)		98 (100.0)	
**Sex**					
Male	206 (26.4)	113 (21.9)	<.001	10 (10.2)	<.001
Female	573 (73.6)	403 (78.1)		88 (89.8)	
**Race/ethnicity**					
White, non-Hispanic	43 (5.5)	22 (4.3)	.001	7 (7.1)	<.001
Black, non-Hispanic	195 (25.0)	113 (21.9)		9 (9.2)	
Hispanic	419 (53.8)	303 (58.7)		73 (74.5)	
Non-Hispanic other/refused	122 (15.7)	78 (15.1)		9 (9.2)	
**Age, y**					
18–24	26 (3.3)	19 (3.7)	.43	5 (5.1)	.007
25–39	252 (32.4)	175 (33.9)		45 (45.9)	
40–64	405 (52.0)	262 (50.8)		41 (41.8)	
≥65	96 (12.3)	60 (11.6)		7 (7.1)	
**Education level**					
High school degree or less	496 (63.7)	359 (69.6)	<.001	76 (77.6)	.02
Some college	138 (17.7)	80 (15.5)		13 (13.3)	
Bachelor’s or more	105 (13.5)	54 (10.5)		7 (7.1)	
Missing or refused	40 (5.1)	23 (4.5)		2 (2.0)	
**Annual household income, $**					
<25,000	415 (53.3)	297 (57.6)	<.001	75 (76.5)	<.001
25,000–49,999	177 (22.7)	102 (19.8)		8 (8.2)	
≥50,000	80 (10.3)	43 (8.3)		2 (2.0)	
Missing or refused	107 (13.7)	74 (14.3)		13 (13.3)	
**Employment status**					
Not employed	282 (36.2)	202 (39.2)	.02	62 (63.3)	<.001
Retired	102 (13.1)	58 (11.2)		7 (7.1)	
Working part-time or full-time	395 (50.7)	256 (49.6)		29 (29.6)	
**SNAP recipient**					
No	451 (57.9)	275 (53.3)	<.001	3 (3.1)	<.001
Yes	328 (42.1)	241 (46.7)		95 (96.9)	
**Usual source for fruits and vegetables**					
Supermarket/grocery store	325 (41.7)	185 (35.9)	<.001	27 (27.6)	<.001
Produce cart	349 (44.8)	263 (51.0)		63 (64.3)	
Farmers’ market or produce store	50 (6.4)	28 (5.4)		1 (3.1)	
Don't know or other	455 (7.1)	40 (7.8)		7 (5.1)	
**Vendor**					
A	178 (22.9)	94 (18.2)	<.001	9 (9.2)	<.001
B	178 (22.9)	178 (34.5)		51 (52.0)	
C	244 (31.3)	244 (47.3)		38 (38.8)	
D	179 (23.0)	—		—	
**How often does respondent shop at this Green Cart?**					
Less than 2 or 3 times/mo	172 (22.1)	90 (17.4)	<.001	14 (14.3)	.01
1–6 times/wk	463 (59.4)	340 (65.9)		72 (73.5)	
At least once/d	144 (18.5)	86 (16.7)		12 (12.2)	
**Data collection round**					
1	341 (43.8)	165 (32.0)	<.001	38 (38.8)	.02
2	174 (22.3)	174 (33.7)		15 (15.3)	
3	264 (33.9)	177 (34.3)		45 (45.9)	
**Were the items purchased to share?**					
No	225 (28.9)	134 (26.0)	.04	16 (16.3)	.009
Yes	550 (70.6)	379 (73.5)		82 (83.7)	
Missing	4 (0.5)	3 (0.6)		0	
**Purchased at least one type of . . .^b^**					
Fruits	629 (80.7)	413 (80.0)	.48	89 (90.8)	.007
Vegetables	341 (43.8)	259 (50.2)	<.001	56 (57.1)	.004
Dark green vegetables	38 (4.9)	22 (4.3)	.27	10 (10.2)	.009
Red or orange vegetables	78 (10.0)	71 (13.8)	<.001	10 (10.2)	.95
Starchy vegetables	45 (5.8)	42 (8.1)	<.001	9 (9.2)	.11
Other vegetables	251 (32.2)	178 (34.5)	.06	47 (48.0)	<.001

Abbreviations: —, not applicable; EBT, electronic benefit transfer; SNAP, Supplemental Nutrition Assistance Program.

a *P* values are for χ^2^ tests of association for subsample versus the rest of the sample.

b Respondents could choose more than one option.

On average, surveyed consumers purchased 6.9 cup equivalents of fruits and 1.8 cup equivalents of vegetables per transaction (Table 2). Customers generally bought more than one type of fruit (1.4) and less than one type of vegetable (0.7). Vegetables identified as “other” (eg, corn, onions, garlic, iceberg lettuce) were most commonly purchased. Dark green (eg, spinach, kale), red or orange (eg, beets, carrots), and starchy (eg, potatoes, parsnips) vegetables represented a minority of the purchases. This rank order was true regardless of the availability of EBT or payment method.

**Table 2:** Summary of Fruit and Vegetable Purchases for Full Sample and Subsamples of Survey Respondents at 4 Green Carts in New York City, 2013 and 2014

Characteristic	Entire Sample (n = 779), Mean No. (SD)	Consumers at Green Carts With EBT (n = 516)		Consumers Who Made Purchases Using SNAP (n = 98)	
		Mean No. (SD)	*P* Value^a^	Mean No. (SD)	*P* Value^a^
**Fruit and vegetable whole cup equivalents** b					
**Total**	8.7 (11.0)	10.4 (12.4)	<.001	18.5 (19.1)	<.001
Fruits	6.9 (9.6)	7.8 (10.8)	<.001	14.8 (17.2)	<.001
Vegetables	1.8 (5.1)	2.6 (6.0)	<.001	3.7 (7.7)	<.001
Dark green vegetables	0.1 (0.8)	0.1 (0.7)	.18	0.1 (0.6)	.88
Red or orange vegetables	0.1 (0.4)	0.1 (0.5)	.002	0.1 (0.4)	.81
Starchy vegetables	0.3 (1.5)	0.4 (1.8)	.006	0.4 (1.4)	.54
Other vegetables	1.4 (4.7)	2.0 (5.6)	<.001	3.2 (7.0)	<.001
**Type of fruit and vegetable purchased**					
Fruits	1.4 (1.2)	1.5 (1.3)	.008	2.4 (1.5)	<.001
Vegetables	0.7 (1.0)	0.9 (1.2)	<.001	1.2 (1.5)	<.001
Dark green vegetables	0.1 (0.2)	0.1 (0.2)	.34	0.1 (0.3)	.01
Red or orange vegetables	0.1 (0.3)	0.2 (0.4)	<.001	0.1 (0.4)	.71
Starchy vegetables	0.1 (0.2)	0.1 (0.3)	<.001	0.1 (0.3)	.12
Other vegetables	0.4 (0.7)	0.5 (0.8)	<.001	0.8 (1.0)	<.001

Abbreviations: EBT, electronic benefit transfer; SNAP, Supplemental Nutrition Assistance Program; SD, standard deviation.

a *P* value for *t* test for association between specified subsample and rest of sample.

b The types and quantity of fruit and vegetable purchases were entered into the Automated Self-Administered 24-hour Recall system (ASA24), a web-based program developed by the National Cancer Institute (13). ASA24 calculated comparable food quantities, or cup equivalents, which are a volume measure of the edible portion of the item that fits in a 1-cup measuring cup (14).

Customers who reported using SNAP benefits at a Green Cart purchased more fruits and vegetables per transaction than customers who paid cash only: 18.5 cup equivalents versus 7.3 cup equivalents (Table 2) (*P* < .001). Larger purchase quantity and variety of “other” vegetables accounted for the total difference in cup equivalents purchased by SNAP versus cash-only–paying survey participants.

Results from negative binomial regressions indicated that the use of SNAP benefits at Green Carts was positively associated with having purchased a larger quantity of fresh produce (Table 3). Controlling for various consumer and design characteristics (sex, age, race/ethnicity, education level, annual household income, employment status, SNAP participation, usual source for produce, frequency of shopping at the sampled Green Cart, intention to share purchased items, data collection round, mobile vendor), the marginal effect of having paid using SNAP benefits was robust across regressions: 5.4 cup equivalents for the full sample (*P* < .001) and 6.4 cup equivalents when the sample was restricted to consumers at Green Carts equipped with an EBT machine (*P* < .001).

**Table 3:** Marginal Effect of Using SNAP on Purchased Cups Equivalents of Fruits and Vegetables at 4 Mobile Produce Carts in New York City, 2013 and 2014^a^

Payment Method	All Survey Respondents (n = 779)						Only Respondents Shopping at Green Carts With an EBT Machine (n = 516)					
	1		2		3		4		5		6	
	Fruit		Vegetable		Total Fruits and Vegetables		Fruit		Vegetable		Total Fruits and Vegetables	
	Δ	*P* Value^b^	Δ	*P* Value^b^	Δ	*P* Value^b^	Δ	*P* Value^b^	Δ	*P* Value^b^	Δ	*P* Value^b^
Cash only	1 [Reference]						1 [Reference]					
SNAP or SNAP and cash	5.0	<.001	0.7	.20	5.4	<.001	5.4	<.001	1.2	.13	6.4	<.001

Abbreviations: —, not applicable; EBT, electronic benefit transfer; SNAP, Supplemental Nutrition Assistance Program.

a Negative binomial regression included controlled for sex, race/ethnicity, age, education level, annual household income, employment status, SNAP participation, usual source for fruits and vegetables, how often respondent shops at this Green Cart, vendor accepts EBT, and whether items were purchased to share.

b *P* values are for a χ^2^ test of constituent contrasts.

Incidence rate ratios (IRRs) for all variables in the negative binomial regressions are shown in the Appendix. Green Cart customers who reported that they intended to share their produce purchased 1.5 times more fruits and vegetables than those who did not intend to share, for both the whole sample and among shoppers just at carts with EBT machines. Among all respondents, people aged 25 to 39 years purchased substantially more vegetables than did those aged 18 to 24 years (IRR = 3.3, *P* = .01). Hispanic survey participants purchased significantly more fruits and vegetables from Green Carts than non-Hispanic white respondents (IRR = 1.3, *P* =.03).

## Discussion

This research builds on our previous work evaluating the possible benefits of expanding the introduction of EBT machines at produce carts (12). In that study, we found that customers who used SNAP benefits at Green Carts spent significantly more per transaction on produce than did those who paid with cash only. Here, we found that the introduction of mobile produce carts, particularly those equipped with EBT machines, in neighborhoods with limited availability of fresh fruits and vegetables could be a positive step toward improving food access. On average, the customers we surveyed at a sample of these mobile vendors purchased nearly 9 cup equivalents of fresh produce per transaction. Notably, fruit represented most of the items purchased from the surveyed mobile produce carts (6.9 of 8.7 cup equivalents purchased per average transaction). We believe this is likely a consequence of the fact that Green Carts generally have more types of fruits than vegetables available for sale.

Although the introduction of EBT machines at mobile produce carts is a promising mechanism for increasing access to fresh produce, there are challenges that may limit the widespread use of EBT machines by mobile produce cart vendors. For example, the EBT machines require an upfront payment of $900 per machine, a $35 monthly fee, and a 3.5-cent charge per transaction. Despite a financial support program to help vendors overcome this barrier (9), less than a third of eligible vendors were equipped with EBT machines by mid-2013 (7). On the basis of brief interviews with the vendors who operated the Green Carts where we conducted data collection, the general sentiment was that EBT machines increased sales and were generally a good investment. However, it is unclear how many vendors would be willing or able to afford the high costs of the machines without financial support. In addition, administrative hurdles may limit the adoption of EBT machines among produce vendors.

We believe the results from our study are noteworthy; however, this study has limitations. First, the sampled carts were all located in one borough of New York City, and surveys were conducted during most, but not all, Green Cart operating hours. Additionally, survey respondents volunteered to participate. Therefore, we do not know the extent to which our sample is representative of all Green Cart customers. Second, although we know whether participants intended to share their produce purchases, we do not have information about how the purchased items were actually shared (ie, how much or with how many people). Third, we did not collect identifying information from our survey participants. Thus, we do not know if there were multiple surveys from the same participant. If there were repeat measures, then we may have underestimated our standard errors. Fourth, we did not conduct a comprehensive inventory of variety, quality, or quantity of produce sold at the Green Carts we observed. As a result, to the extent that the selection of produce at mobile produce vendors varies across vendor and time, our results may not generalize beyond our research context. Finally, we lack comprehensive purchasing information for our survey respondents (ie, quantity of all fresh produce purchases made from other retailers). In light of these limitations, we cannot make any causal assertions about whether SNAP beneficiaries purchased more fruits and vegetables in total, nor whether the availability of EBT machines at Green Carts influenced where consumers chose to shop.

Adequate consumption of minimally processed fruits and vegetables is an important component of a healthy diet and is associated with an array of positive health outcomes (15,16). Although the research on the associations between food access and health outcomes is mixed (17,18), we are encouraged by our findings and would like to see rigorous causal research on the effect of access to mobile produce carts on fruit and vegetable consumption and overall diet quality. Results from such research could advance the literature on the role of this type of intervention in improving public health.

EBT capabilities may increase fresh fruit and vegetable purchases, helping to overcome a barrier to a healthy diet. Although these findings are not informative about total consumer fruit and vegetable procurement, the results from our analysis suggest that the availability of EBT machines at produce carts may increase purchases of fresh fruits and vegetables from those carts. The provision of mobile produce cart permits and financial assistance for EBT machines could be sustainably scalable, although we invite additional causal research on the subject before making policy recommendations.

## Appendix Negative Binomial Incident Rate Ratios for Cup Equivalents of Fruit and Vegetable Purchases at 4 Mobile Produce Carts in New York City, 2013 and 2014

**Table:**

Payment method	All Survey Respondents (n = 779)						Only Respondents Shopping at Green Carts With an EBT Machine (n = 516)					
	1		2		3		4		5		6	
	Fruit		Vegetable		Total fruits and vegetables		Fruit		Vegetable		Total fruits and vegetables	
	IRR	*P* ^a^	IRR	*P* ^a^	IRR	*P* ^ a^	IRR	*P* ^a^	IRR	*P* ^a^	IRR	*P* ^a^
**Cash only**	1 [Reference]						1 [Reference]					
SNAP or SNAP and cash	1.8	<.001	1.4	.15	1.7	<.001	1.8	<.001	1.5	.08	1.7	<.001
**Sex**												
Male	1 [Reference]						1 [Reference]					
Female	1.0	.77	1.3	.18	1.1	.36	1.1	.64	1.3	.30	1.1	.16
**Race/ethnicity**												
White, non-Hispanic	1 [Reference]						1 [Reference]					
Black, non-Hispanic	1.0	.85	1.4	.48	1.0	.83	0.7	.15	2.0	.18	0.9	.55
Hispanic	1.2	.17	1.6	.27	1.3	.03	1.0	.83	3.1	.02	1.3	.11
Non-Hispanic other/refused	1.0	.84	0.9	.90	1.1	.69	0.9	.48	1.9	.2	1.1	.78
**Age, y**												
18–24	1 [Reference]						1 [Reference]					
25–39	1.0	.87	3.3	.01	1.2	.32	1.2	.50	5.1	<.001	1.6	.03
40–64	0.9	.53	2.1	.09	1.0	.81	0.9	.78	4.0	<.001	1.2	.28
≥65	0.9	.60	3.0	.05	1.1	.61	0.9	.61	6.8	<.001	1.4	.19
**Education level**												
≤High school degree	1 [Reference]						1 [Reference]					
Some college	0.9	.24	0.9	.71	0.9	.22	0.8	.18	1.1	.82	0.9	.03
Bachelor’s or more	1.2	.27	1.4	.23	1.2	.11	0.9	.72	1.3	.34	1.0	.28
Missing or refused	1.0	.85	2.3	.03	1.1	.74	1.2	.51	1.6	.17	1.2	.19
**Annual household income, $**												
<25,000	1 [Reference]						1 [Reference]					
25,000–49,999	0.8	.08	0.6	.04	0.8	.02	0.9	.28	0.7	.09	0.8	.10
≥50,000	1.1	.52	0.5	.05	1.0	.83	1.0	.88	0.5	.10	0.8	.18
Missing or refused	0.9	.40	0.6	.02	0.9	.14	0.9	.24	0.7	.15	0.9	.14
**Employment status**												
Not employed	1 [Reference]						1 [Reference]					
Retired	0.9	.38	1.0	.99	0.9	.53	0.9	.44	0.9	.81	0.9	0.45
Working part-time or full-time	1.0	.96	1.1	.73	1.0	.57	0.9	.35	1.2	.38	1.0	0.67
**SNAP recipient**												
No	1 [Reference]						1 [Reference]					
Yes	1.0	.79	0.8	.24	0.9	.38	0.9	.23	0.8	.36	0.8	0.03
**Usual source for fruits and vegetables**												
Supermarket or grocery store	1 [Reference]						1 [Reference]					
Produce cart	1.1	.54	0.9	.64	1.1	.40	1.0	.90	0.9	.69	1.0	0.86
Farmers’ market or produce store	1.3	.1	0.5	.07	1.2	.28	1.3	.19	0.7	.4	1.1	0.39
Don't know or other	1.0	.78	1.0	.90	1.2	.27	1.0	.93	1.2	.64	1.2	0.23
**Vendor**												
A	1 [Reference]						1 [Reference]					
B	1.1	.6	2.0	.10	1.3	.08	1.0	.90	2.2	.02	1.3	0.14
C	0.7	.01	0.8	.50	0.7	.01	0.7	.02	0.9	.60	0.7	0.02
D	0.9	.55	<.1	<.001	0.7	.03	NA	NA	NA	NA	NA	NA
**How often does respondent shop at this Green Cart?**												
Less than 2 or 3 times/mo	1 [Reference]						1 [Reference]					
1–6 times/wk	0.9	.48	1.3	.24	0.9	.49	1.1	.61	1.2	.36	1.1	0.51
At least once/d	0.7	.01	1.7	.06	0.8	.11	0.9	.30	1.8	.07	1.0	0.94
**Vendor accepts EBT**												
No	1 [Reference]						NA	NA	NA	NA	NA	NA
Yes	1.4	.13	1.5	.32	1.3	.11	NA	NA	NA	NA	NA	NA
**Items purchased to share**												
No	1 [Reference]						1 [Reference]					
Yes	1.5	<.001	1.8	<.001	1.5	<.001	1.5	<.001	1.6	<.001	1.5	<.001
**Data collection round**												
1	1 [Reference]						1 [Reference]					
2	0.7	.01	1.6	.08	0.8	.07	0.6	<.001	1.7	.05	0.7	0.01
3	0.6	<.001	0.9	.66	0.7	<.001	0.5	<.001	0.9	.55	0.5	<.001
Constant	0.8	<.001	3.7	<.001	0.5	<.001	0.9	.10	3.4	<.001	0.5	<.001

Abbreviations: EBT, electronic benefit transfer; IRR, incidence rate ratio; NA, not applicable; SNAP, Supplemental Nutrition Assistance Program.

^a^ *P* values are for *z* test of the null hypothesis that the IRR is 1, given that the rest of the predictors are in the model.

## References

[1] Eng M . Fruit and vegetable stands to sprout in Chicago. Chicago Tribune; 2012. http://articles.chicagotribune.com/2012-06-06/news/ct-met-food-stand-ordinance-20120607_1_licenses-food-deserts-fruit-and-vegetable. Accessed February 8, 2016.

[2] Setters A. Mobile vendors can sell produce in neighborhoods where it’s grown: pilot program puts fresh produce in food deserts. WLWT; 2013. http://www.wlwt.com/news/local-news/cincinnati/mobile-vendors-can-sell-produce-in-neighborhoods-where-its-grown/20467794. Accessed February 8, 2016.

[3] Cannon J . Memphis mobile market creates oasis in food desert. USA Today; 2015. http://college.usatoday.com/2015/03/27/memphis-mobile-food-market-creates-oasis-in-food-desert/. Accessed February 8, 2016.

[4] New York City Press Release. Mayor Bloomberg signs legislation establishing 1,000 new “Green Cart” permits. News from the Blue Room, PR-086-08. New York; 2008. http://tinyurl.com/yf4yo4m.

[5] Leggat M , Kerker B , Nonas C , Marcus E . Pushing produce: the New York City Green Carts initiative. J Urban Health 2012;89(6):937–8. 10.1007/s11524-012-9688-4 22684423 PMC3531353

[6] Li KY , Cromley EK , Fox AM , Horowitz CR . Evaluation of the placement of mobile fruit and vegetable vendors to alleviate food deserts in New York City. Prev Chronic Dis 2014;11:140086. 10.5888/pcd11.140086 25211506 PMC4164039

[7] Fuchs ER , Holloway SM , Feathers A , Fuchs ER , Holloway SM . An evaluation of the New York City Green Cart Initiative to expand access to healthy produce in low-income neighborhoods. Columbia Univ Sch Int Aff Case Study Ser Glob Public Policy 2014;2(2).

[8] New York State Council on Food Policy. Current initiatives, accomplishments and collaborative efforts: 2013 annual report; 2013. http://www.agriculture.ny.gov/CFP2/NYSCFP_Report_2013.pdf. Accessed July 6, 2017.

[9] Green cart implementation: year one. New York (NY): Citizen’s Committee for Children of New York; 2010.

[10] Lucan SC , Maroko A , Shanker R , Jordan WB . Green Carts (mobile produce vendors) in the Bronx — optimally positioned to meet neighborhood fruit-and-vegetable needs? J Urban Health 2011;88(5):977–81. 10.1007/s11524-011-9593-2 21691925 PMC3191209

[11] Kerker B , Farley S , Johns M , Leggat P , Nonas C , Parton H . Green Cart Evaluation, 2008–2011. https://www1.nyc.gov/assets/doh/downloads/pdf/epi/databrief48.pdf. Accessed July 6, 2017.

[12] Breck A , Kiszko KM , Abrams C , Elbel B . Spending at mobile fruit and vegetable carts and using SNAP benefits to pay, Bronx, New York, 2013 and 2014. Prev Chronic Dis 2015;12:140542. 10.5888/pcd12.140542 26043302 PMC4456853

[13] 24-Hour Recall (Beta version; FNDDS 1.0) (ASA24). Bethesda (MD): National Cancer Institute; 2009.

[14] Stewart H . Fruit and vegetable recommendations can be met for $2.10 to $2.60 per day; 2016. http://www.ers.usda.gov/amber-waves/2016-march/fruit-and-vegetable-recommendations-can-be-met-for-$210-to-$260-per-day.aspx#.V3QTrtIrJhF. Accessed June 29, 2016.

[15] Cavallo DN , Horino M , McCarthy WJ . Adult intake of minimally processed fruits and vegetables: associations with cardiometabolic disease risk factors. J Acad Nutr Diet 2016;116(9):1387–94. 10.1016/j.jand.2016.03.019 27174619 PMC5034720

[16] Bertoia ML , Mukamal KJ , Cahill LE , Hou T , Ludwig DS , Mozaffarian D , Changes in intake of fruits and vegetables and weight change in United States men and women followed for up to 24 years: analysis from three prospective cohort studies. PLoS Med 2015;12(9):e1001878. 10.1371/journal.pmed.1001878 26394033 PMC4578962

[17] Handbury J , Rahkovsky I , Schnell M . What drives nutritional disparities? Retail access and foods purchases across the socioeconomic spectrum. NBER Working Paper 21126; 2015. Report No.: 21126.

[18] Lee H . The role of local food availability in explaining obesity risk among young school-aged children. Soc Sci Med 2012;74(8):1193–203. 10.1016/j.socscimed.2011.12.036 22381683

